# Reablement in community-dwelling adults: study protocol for a randomised controlled trial

**DOI:** 10.1186/1471-2318-14-139

**Published:** 2014-12-18

**Authors:** Hanne Tuntland, Birgitte Espehaug, Oddvar Forland, Astri Drange Hole, Egil Kjerstad, Ingvild Kjeken

**Affiliations:** Centre for Care Research Western Norway, and Department of Occupational Therapy, Physiotherapy and Radiography, Bergen University College, P.O. Box 7030, 5020 Bergen, Norway; Centre for Evidence-based Practice, Bergen University College, P.O. Box 7030, 5020 Bergen, Norway; Centre for Care Research Western Norway, Bergen University College, P.O. Box 7030, 5020 Bergen, Norway; Haraldsplass Deaconess University College, Ulriksdal 10, 5009 Bergen, Norway; Bergen University College, P.O. Box 7030, 5020 Bergen, Norway; Uni Research Rokkan Centre, P.O. Box 7810, 5020 Bergen, Norway; Diakonhjemmet Hospital, National Advisory Unit on Rehabilitation in Rheumatology, P.O.Box 23, Vinderen, 0319 Oslo, Norway; Program of Occupational Therapy, Prosthetics and Orthotics, Oslo and Akershus University College of Applied Sciences, P.O. Box 4, St. Olavs plass, 0130 Oslo, Norway

**Keywords:** Activities of daily living, Rehabilitation, Aged, Randomised controlled trial, Home-care services, Health care costs

## Abstract

**Background:**

As a result of the ageing population, there is an urgent need for innovation in community health-care in order to achieve sustainability. Reablement is implemented in primary care in some Western countries to help meet these challenges. However, evidence to support the use of such home-based rehabilitation is limited. Reablement focuses on early, time-intensive, multidisciplinary, multi-component and individualised home-based rehabilitation for older adults with functional decline. The aim of this study is to investigate the effectiveness of reablement in home-dwelling adults compared with standard treatment in relation to daily activities, physical functioning, health-related quality of life, use of health-care services, and costs.

**Methods/Design:**

The study will be a 1:1 parallel-group randomised controlled superiority trial conducted in a rural municipality in Norway. The experimental group will be offered reablement and the control group offered standard treatment. A computer-generated permuted block randomisation sequence, with randomly selected block sizes, will be used for allocation. Neither participants nor health-care providers will be blinded, however all research assistants and researchers will be blinded. The sample size will consist of 60 participants. People will be eligible if they are home-dwelling, over 18 years of age, understand Norwegian and have functional decline. The exclusion criteria will be people in need of institution-based rehabilitation or nursing home placement, and people who are terminally ill or cognitively reduced. The primary outcome will be self-perceived performance, and satisfaction with performance of daily activities, assessed with the Canadian Occupational Performance Measure. In addition, physical capacity, health-related quality of life, use of health-care services, and cost data will be collected at baseline, and after 3 and 9 months in both groups, and again after 15 months in the intervention group. Data will be analysed on an intention-to-treat basis using a linear mixed model for repeated measures.

**Discussion:**

The findings will make an important contribution to evaluating cost-effective and evidence-based rehabilitation approaches for community-dwelling adults.

**Trial registration:**

The trial was registered in ClinicalTrials.gov November 20, 2012, identifier: NCT02043262.

## Background

The increasing aged population, in conjunction with an expected shortage of health-care personnel in developed countries, present a huge challenge to the containment of future health-care costs [1]. Further, in times of budget cuts to front-line public services, policy makers are seeking new approaches to get more for less by investing the resources available in ways which have an optimal impact on outcomes [2]. As a result, in recent years, there has been an increasing interest in home-care reablement services (hereafter ‘reablement’) [3]. Reablement, termed ‘restorative care’ in US, Australia and New Zealand, is an approach to improve home-care services for older people needing care or experiencing functional decline. It is a goal-directed and intensive intervention, which takes place in the person’s home and local surroundings with a focus on enhancing performance of everyday activities defined as important by the person. The intention is to increase independence in daily activities, and enable people to age in place, be active and participate socially and societally. The health-care providers are organised in an integrated, coordinated multidisciplinary team that works together with the person towards shared goals [4]. In Norway, a substantial proportion of municipalities are currently implementing reablement.

The effects of reablement have so far been evaluated in three randomised controlled trials (RCTs). Two of these were conducted in New Zealand, and the results showed improved social support and physical functioning [5] and improved quality of life [6]. In a third Australian RCT with 750 participants and a 2-year follow-up, reablement was compared with usual care [7]. Even if the results showed few differences between groups in individual outcomes over time, a significantly smaller proportion of the reablement group required assistance with personal care. In a later publication from the same trial, the results showed that participants in the reablement group required fewer home-care hours, were less likely to be approved for a higher level of aged care such as nursing homes, and were less likely to be in need of emergency department treatment than the conventional care group [8]. The results thereby indicate that reablement may reduce the need for ongoing home-care, as well as for other health care services.

Two studies have investigated cost-effectiveness of reablement. The results in a large British non-randomised study with 1015 participants showed no significant differences between the intervention and control groups with respect to cost savings [9]. However, the results in the aforementioned Australian RCT showed that aggregated health and home-care costs of reablement were lower than the costs of the conventional home-care [8].

In summary, the research on the effectiveness of reablement is scarce, the results are conflicting and more studies are needed.

### Aims and research questions for the study

The main objective will be to evaluate health effects and cost-effectiveness of a reablement intervention compared with current standard treatment for home-dwelling adults experiencing functional decline.

More specifically, our study will answer the following research questions:

- Is reablement more effective with regard to performance and satisfaction with performance of daily activities, physical functioning, and health-related quality of life compared with standard treatment?
- Does the experimental intervention or the control intervention provide more cost-effective use of health-care resources?

## Methods/Design

### Study design and setting

**Figure 1:**
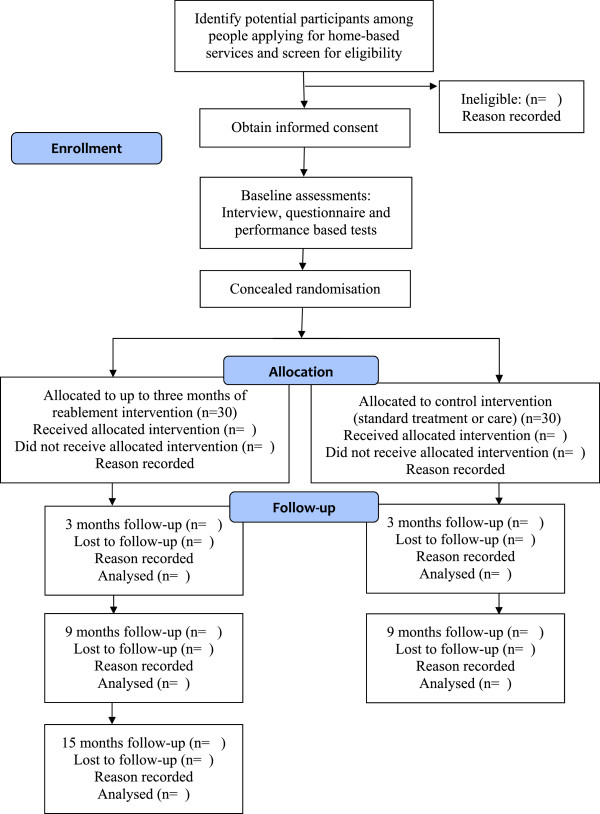
**Flow diagram of study protocol.**

This will be a parallel-group randomised controlled superiority trial in which all participants will be assessed at baseline, and after 3 and 9 months. Participants in the intervention group will also be re-assessed after 15 months. The study will be conducted in a primary care setting in a rural municipality in Norway with approximately 14,000 inhabitants. The intervention group will receive reablement and the control group will receive standard treatment and care. For ethical reasons, the control group will be offered reablement 9 months after baseline assessment. Thus, potential long-term effects data at 15 months after baseline will only be collected from the experimental group. The flow diagram of the study protocol is outlined in Figure 1. The protocol employs relevant standard protocol items for clinical trials according to the SPIRIT 2013 statement [10], and follows the CONSORT statement [11] for transparent reporting. The trial is registered in ClinicalTrials.gov, identifier NCT02043262.

### Participants and eligibility criteria

People applying for, or referred to, home-based services are potential participants in the study. Health-care providers in a central office responsible for the allocation of public health-services in the municipality will identify potential participants amongst the applicants, inform them about the new reablement service, and invite them to participate. Those who are interested will be screened for eligibility, and in order to enrol, participants will have to give their written informed consent. An additional strategy to achieve adequate participant enrolment to reach target sample size will be self-selection through advertisements.

We will include home-dwelling persons over the age of 18 years, who currently live in the municipality, are able to understand Norwegian, and have a functional decline in one or more daily activities. To enhance recruitment, the study will not be restricted to older adults even though we expect the majority of participants to be in that age group. We will exclude participants if they are in need of institution-based rehabilitation or a nursing home placement, are terminally ill, or are moderately or severely cognitively reduced (subjectively assessed by health-care providers based on observation and communication).

### Randomization and allocation concealment

A bio-statistician (BE), not involved in the assignment of participants to groups, will perform the randomisation with an allocation ratio of 1:1 using a computer-generated permuted block randomisation sequence, with randomly selected block sizes. We will conceal the allocation sequence in sequentially numbered, opaque, sealed envelopes. The allocation list will be stored in a safe deposit box in a central office in the municipality. After baseline assessments, but still in the home of the participant, the research assistant will randomly assign the participant to one of the two trial groups, by means of calling the central office. The health-care provider in this office will unlock the safe deposit box containing the allocation list and reveal information on the particular participant’s group assignment. To prevent subversion of the allocation sequence, the name of the participant will be written on the envelope after disclosing group assignment in each case. Hence, neither health-care providers enrolling participants nor research assistants will have influence on group assignment.

### Blinding

Occupational therapists and physiotherapists in the municipality will conduct the baseline assessments in the participant’s home prior to randomisation. The research assistants, who are also occupational therapists and physiotherapists, will be blinded to group allocation and perform all follow-up assessments. The research assistants will urge the participants not to reveal their group allocation during follow-up assessments, which will also take place in the participant`s home. The success of research assistant blinding will be evaluated for both follow-ups. Due to the nature of the interventions, it will not be possible to blind participants and health-care providers. Researchers performing data entry and data analysis will, however, be blinded to group allocation.

### Training of the intervention providers

Reablement will be implemented in the municipality after a period of administrative planning and competence-building. The competence-building will involve all the members of the multidisciplinary reablement team, such as nurses, auxiliary nurses, social educators, occupational therapists, physiotherapists, home-helpers and assistants. The health-care providers will be given lectures and seminars, and invited to attend external courses. Special attention will be given to the use of the Canadian Occupational Performance Measure (COPM) [12], a patient-specific measure which will be used to identify activity limitations and as a basis for formulating the goals that will be addressed in the reablement intervention. It will also be important to ensure that all members of the reablement team have internalised the required rehabilitation approach of encouraging the participant to self-management.

### Interventions

#### Reablement

The intervention will have a maximum duration of 3 months. As part of baseline assessments, the occupational therapist and physiotherapist will use the COPM interview to identify activity limitations perceived as important by the participant. This information will thereafter be used to develop a rehabilitation plan, and to ensure congruence between the participant’s needs, therapy priorities, and interventions. After initiating the reablement intervention, the occupational therapist and physiotherapist will supervise the home-care personnel, some of whom have no formal education, in how to encourage and assist the person in the daily training. The focus is on stimulating the participants to do the daily tasks themselves, rather than receiving help or letting others do the tasks for them. As reablement is tailored according to participants’ goals, the components of the invention will vary. However, the intervention will consist of both general and individual features as described in Table 1.

**Table 1:** **Features of the reablement intervention**

General features	Individual features
• The rehabilitation period will be a maximum of 3 months.	• *Training in daily activities* such as dressing, food preparation, vacuuming, bus transport, visiting friends at a club, or being able to knit.
• An occupational therapist or physiotherapist will conduct the COPM interview and develop the rehabilitation plan together with the participant based on the identified activity goals. Thereafter, an integrated multidisciplinary team with shared goals will guide the participant during the whole rehabilitation period.	• *Adaptations* such as advice on appropriate assistive technology or adapting the activity itself or the environment, in order to simplify activity performance.
• In addition to home-care personnel assisted training, a minimum of one hour physiotherapist and/or occupational therapist assisted training will be guaranteed each week.	• *Exercise programs* such as indoor or outdoor walking with or without walking aids, climbing stairs, transferring, and performing exercises to improve strength, balance or fine motor skills. The exercises will be incorporated into daily routines and the person will be given a manual explaining each of the exercises and encouraged to train on their own.
• The treatment will involve repetitive training and multiple home-visits by health-care personnel, who will be present during daily training for the purposes of building confidence and relearning skills.	
• All health-care personnel will stimulate the participant in self-management and self-training.	

#### The control intervention

Standard treatment/care is the conventional treatment homebound persons in most municipalities in Norway are offered, and this will be used as the comparator. For most participants, standard treatment will involve receiving the compensating help they apply for, in terms of personal or practical assistance, Meals on Wheels, safety alarm or assistive technology. However, for some participants, it may comprise rehabilitation by an occupational therapist and/or physiotherapist based on the participants’ own efforts. Hence, the standard treatment will also be diverse. The standard treatment will not be time-limited, and may continue after 3 months if needed.

### Outcomes

Data collection will involve the use of four different outcomes measures. In addition, cost outcomes in terms of consumption of different home-based services will be registered on a daily basis during the first 9 months after inclusion. This comprises registering minutes spent by different health professionals in the participant’s home. The first author will train all research assistants in how to conduct the data collection in order to obtain protocol adherence. Table 2 provides an overview of the various outcomes that will be measured.

**Table 2:** **Summary of measures to be collected**

Outcome	Data collection instrument and scale	Time points
**Primary outcome measures**		
Activity performance	Canadian Occupational Performance Measure. Scale 1–10, 1 is low performance	t1, t2, t3
Satisfaction with activity performance	Canadian Occupational Performance Measure. Scale 1–10, 1 is low satisfaction	t1, t2, t3
**Secondary outcome measures**		
Lower extremity function and mobility	Timed Up and Go, measured in seconds, the second of two trials will be used	t1, t2, t3
Grip strength	Jamar dynamometer, measured in kilograms, the mean of two trials will be used	t1, t2, t3
Health-related quality of life	COOP/Wonka. Scale 1–5, 1 is low health-related quality of life	t1, t2, t3
**Other measures**		
Age	Years	t1
Gender	Female/Male	t1
Marital status	Married/Cohabiting/Single/Widowed/Separated or divorced	t1
Level of education	Primary school/High school/1–3 years university/> 4 years university	t1
History of paid work	Yes/No	t1
Profession	Type of work	t1
Current work status	Retired/Disability benefit/Working	t1, t2, t3
Motivation for rehabilitation	Numeric scale 1–10, 1 is lowest	t1
Main disease	Type of dominant disease	t1, t2, t3
Comorbidity	Presence of additional diseases	t1, t2, t3
Prescribed medication	Type and usage	t1, t2, t3
Un-prescribed medication	Type and usage	t1, t2, t3
Research assistant identification of participant’s group assignment	Yes/No	t2, t3
Control of research assistant’s identification of group assignment of participant	Intervention group/Control group	t2, t3
**Health-care services and cost measures**		
Warranted community-based assistance in time of inclusion	*Type of assistance wanted*	t1
	Home-helper/Nurse/Auxiliary nurse/Occupational therapist/Physiotherapist/Nursing home placement long term/Nursing home placement short term/Assisted living/Meals on Wheels/Safety alarm/Rehabilitation	
Inpatient and outpatient treatment since last assessment	*Frequency and type of co-interventions*	t2, t3
	Hospital admissions/Admissions to other institutions/Day centre placement/Outpatient treatment	
Current home-based assistance offered	*Presence and frequency of home-based assistance*	t2, t3, t4
	Home-helper/Nurse/Auxiliary nurse/Occupational therapist/Physiotherapist/Meals on Wheels/Reablement/No assistance	
Current community institution-based service offered	*Type of institution-based service offered*	t4
	Nursing home placement long-term/Nursing home placement short-term/Day placement/Other institution placement/No institution placement	
Usage of home-based services	*Daily time registration in minutes of working time used during home-visits*	t5
	Home-helper/Nurse/Auxiliary nurse/Occupational therapist/Physiotherapist/Social educator/Assistant/Speech therapist/Student	

t1 = baseline assessment, t2 = 3 months after baseline assessment, t3 = 9 months after baseline assessment, t4 = 15 months after baseline assessment, t5 = daily assessment during 9 months after baseline assessment.

#### Primary outcome

The primary outcome will be performance of activities of daily living and satisfaction with that performance, measured by the COPM [12]. During a semi-structured interview, the participant will be encouraged to identify problems with their self-care, productivity and leisure activities. The participant will thereafter rate importance of each identified activity on a 1 to 10-point scale, before the five most important activities are rated for performance and satisfaction with performance, again on 1 to 10-point scales (higher scores reflect higher importance, better performance or higher satisfaction). A change of two points is regarded as a clinically relevant improvement or deterioration [12].

A literature review based on 19 methodological studies [13], concludes that COPM is a valid, reliable, clinically useful and responsive outcome measure. The Norwegian version of COPM has been tested for validity and responsiveness [14] and reliability [15] in persons with rheumatic diseases with good results. Psychometric properties have also been found satisfactory in elderly persons with a variety of diagnoses [14, 16, 17].

#### Secondary outcomes

Functional mobility will be measured using the Timed Up and Go (TUG) Test, which was developed as a short test of basic mobility skills in frail community-dwelling elderly persons [18]. The participant will be encouraged to walk fast without compromising safety. The time taken to rise from a chair with arm rests, walk 3 m, cross a line on the floor, turn, walk back, and sit down again will be registered. Normative data for home-dwelling older adults exists [19]. Test-retest reliability [19] and intrarater reliability [18] in community-dwelling elderly people has been found to be excellent and moderate, respectively. Criterion validity [18] and construct validity [20] has also been found to be excellent and moderate, respectively, in a community-dwelling older population.

Grip strength will be measured with the hydraulic instrument, Jamar Dynamometer. The participant will sit in front of a table holding the dynamometer. With the elbow at 90 degrees flection, the participant will be asked to grip and squeeze the dynamometer as hard as possible. Both hands will be tested twice. The mean of the two assessments will be calculated. Normative values for average grip strength in an elderly population are available [21]. The instrument has been tested for criterion validity in a normal population [22] and test-retest reliability in community-dwelling older adults [23] with excellent results.

Health-related quality of life will be measured by the COOP/Wonka, which is a generic, self-reported outcome measure [24]. The chosen revised version [25] consists of six questions with associated drawings, where each question represents a separate domain. The responses are scored on a five-point ordinal scale ranging from 1 to 5 (1 = best, 5 = worst). In a structured review, COOP/Wonka was found to have weak evidence of reliability, adequate evidence of validity, and good evidence of responsiveness in an elderly population [26].

### Sample size calculation

In an earlier study performed on older adults, the standard deviation for the primary outcome has been shown to be 1.4 for COPM performance and 1.6 for COPM satisfaction [27]. With a conservative estimate of the standard deviation of 2.5, sample size calculations showed that 21 participants need to be included in each group to detect a change of 2 points as statistically significant (with a two-sided 5% level and a power of 80%) [28]. We also assumed a within-subject correlation coefficient for the three follow-up measurements of 0.7. To take into account the possibility of a relatively high dropout rate (up to 40%) due to frail participants, 60 participants (30 people in each group) will be included.

### Statistical analysis

A bio-statistician blinded to group allocation will monitor the data analysis. The intention-to-treat principle will be followed.

#### Descriptive statistics

Descriptive statistics of baseline characteristics, and of outcome measures at all time-points, will be presented for each group. Mean (standard deviation), median values (inter-quartile range), or number and percentages will be reported.

#### Analysis of effectiveness

Outcome measures will be compared between the treatment groups at the 3 and 9 month follow-ups using linear mixed-effects models with adjustment for baseline measurements [29]. The mixed-effects model approach (also called random coefficient model or multilevel model) will be used to account for correlated data introduced by the repeated measures study design, due to its versatility in the modelling of the time factor and in allowing varying numbers of measurements per individual. In the analyses, group and time by group interaction will be entered as fixed factors, time as a repeated factor and participant as a random factor. In the case of group imbalance in the distribution of gender or other baseline characteristics, these variables will be included in the model. Models will be fitted with random intercepts and also with random slopes. Robustness and underlying assumptions will be investigated. Estimated regression coefficients will be presented with 95% confidence intervals and p-values.

#### Health economic analysis

To assess potential welfare effects of the intervention, a cost-efficiency analysis (CEA) and a cost-utility analysis (CUA) will be conducted. Effect measures of the intervention are changes in COPM for the CUA and changes in e.g. grip strength for the CEA. Detailed registration of time spent at each home will enable us to establish the aggregate costs associated with provision of services for individuals in both groups. The hourly wage including pay roll tax and other taxes for the different categories of staff members will be applied. Costs will vary according to the duration and/or type of competence that is offered to each participant. The detailed time registration will make it possible to differentiate between types of staff with respect to costs. An incremental cost efficiency ratio (ICER) will be calculated.

Potential long-term changes in the intervention group will be examined after 15 months. Employing the panel data structure, variations in cost per unit change in effect measures across individuals will be analysed, controlling for gender, age, and other variables. Both fixed effect and random effect models will be estimated.

### Ethics and dissemination

The Regional Committees for Medical and Health Research Ethics (REK West, 2012/295) granted ethics approval for the study. The research will be carried out according to the Declaration of Helsinki. Personal confidentiality will be assured and a declaration of voluntary participation with information about the study purposes and consequences, emphasising the right to withdraw from the study, will be signed by each participant. The randomisation procedure is regarded as ethically acceptable, as none of the participants will receive an intervention that is below the standard she or he would otherwise have received if not participating in the trial. Besides, the control group will be offered reablement after completing 9 months follow-up. Thus, delivering an inferior rehabilitation intervention will be avoided.

We will communicate the results in peer-reviewed journals. In addition, results will be presented to health-care professionals and the public through various regional and national events and websites.

## Discussion

To our knowledge, this will be the first RCT examining the effect of reablement in a Scandinavian context. The protocol has been developed according to the SPIRIT 2013 statement [10], follows the CONSORT statement [11], and is registered in ClinicalTrials.gov, 2012/295. Reablement has evolved in countries like Sweden, Denmark and Norway in recent years and is increasingly being implemented in these countries. However, so far, only one Danish non-controlled study, evaluating if a home-based reablement program influenced the ability of older adults to perform activities of daily living, has been published in Scandinavia [30]. Current evidence from international reablement is also sparse and inconclusive [5–9] and evidence from high quality RCTs is lacking. This paper outlines the protocol for a study where the main aim is to assess the effects of reablement on a long-term basis. The trial uses a randomised controlled design, which is considered the gold standard for testing the effect of a specific intervention.

In this trial, a combination of a patient-specific measure (COPM), standardised generic measures (TUG, Jamar Dynamometer), and a questionnaire with standardised items (COOP/Wonka) will be used. The intention is that the combination of instruments will capture the multi-component nature of the experimental intervention and the effects it has on the ability to perform daily activities, functional capacity and health-related quality of life. This will also allow for comparison of populations and results across studies. In addition, the study will provide socio-demographic, health-care service consumption, and related cost data. Thus, despite the modest sample size, it will be a comprehensive study with the potential to capture a diversity of outcomes.

One limitation in the study will be the lack of blinding of participants and health-care providers. We will, however, record and evaluate the success rate of the assessor blinding strategy. Another possible limitation will be the risk of contamination from the intervention arm of the study to the control arm. Due to potential problems with recruitment in a rather sparsely inhabited municipality, the intervention will be implemented in all home-care districts in the municipality. Hence, it will not be possible to avoid the same health-care personnel providing both the experimental and control interventions, even though this will be to different participants. As a consequence, the differences between the groups may be diminished.

A third limitation may be the nature of the COPM interview, which, in a previous study, was found to have a therapeutic effect independent of further interventions [31]. In the COPM interview and scoring process, the participants in both groups will be encouraged to verbalise important activity limitations and participation restrictions. This may have an effect that results in perceptual and behavioural changes initiated by the participant, which again may blur the effects of the reablement intervention. On the other hand, reablement is a goal-directed and individualised intervention. The use of COPM will allow each participant to choose and rate the activity limitations that he/she considers important, thereby capturing aspects of everyday life that are of direct concern to the individual. As a consequence, the “noise” related to items in standardised instruments experienced as irrelevant by participants will be reduced, which in theory will have the potential to make the COPM more responsive to capturing the effects of reablement. In addition, the described activities will be used as a basis for discussing both long-term and short-term goals for reablement, thus enhancing communication and an active role for the participant in the reablement process.

In conclusion, this study will contribute to the knowledge of the effect and cost-effectiveness of reablement in community-dwelling adults.

## Abbreviations

CEA: Cost-efficiency analysis
CONSORT: Consolidated standards of reporting trials
COPM: Canadian occupational performance measure
CUA: Cost-utility analysis
ICER: Incremental cost efficiency ratio
RCT: Randomised controlled trial
SPIRIT: Standard protocol items: recommendations for interventional trials
TUG: Timed up and go.
